# High-fat diet-fed ovariectomized mice are susceptible to accelerated subcutaneous tumor growth potentially through adipose tissue inflammation, local insulin-like growth factor release, and tumor associated macrophages

**DOI:** 10.18632/oncotarget.27832

**Published:** 2020-12-08

**Authors:** Jackie Bader, Meredith Carson, Reilly Enos, Kandy Velazquez, Alexander Sougiannis, Udai Singh, William Becker, Mitzi Nagarkatti, Daping Fan, Angela Murphy

**Affiliations:** ^1^Department of Pathology, Microbiology, & Immunology, School of Medicine, University of South Carolina, Columbia, SC 29209, USA; ^2^Department of Medicine, University of Virginia Health Systems, Charlottesville, VA 22908, USA; ^3^Department of Cell Biology and Anatomy, School of Medicine, University of South Carolina, Columbia, SC 29209, USA

**Keywords:** colorectal cancer, obesity, metabolism, macrophage, inflammation

## Abstract

Background: The association between obesity and colorectal cancer (CRC) risk has been well established. This relationship appears to be more significant in men than in women, which may be attributable to sex hormones. However, controlled animal studies to substantiate these claims and the mechanisms involved are lacking.

Materials and Methods: MC38 murine colon adenocarcinoma cells were injected subcutaneously into high-fat diet (HFD) fed male, female and ovariectomized (OVX) female C57BL/6 mice.

Results: HFD increased tumor growth (main effect) that was consistent with metabolic perturbations (*P* < 0.01). HFD OVX mice exhibited the most significant tumor growth compared to HFD male and female mice (*p* < 0.05) and this was associated with increased subcutaneous adipose tissue (*p* < 0.05). Further, the subcutaneous adipose tissue depots within HFD OVX mice exhibited more severe macrophage associated inflammation compared to female (*P* < 0.01), but not male mice. Conditioned media from subcutaneous adipose tissue of HFD OVX contained higher IGF-1 levels compared to male (*P* < 0.01), but not female mice. Finally, HFD OVX mice had increased M2-like gene expression in their tumor-associated macrophages (TAMs) compared to female mice (*P* < 0.01).

Conclusions: This work provides evidences suggesting adiposity, adipose specific IGF-1, macrophage associated adipose inflammation, and TAMs as potential mechanisms driving obesity-enhanced CRC in females lacking ovarian hormones.

## INTRODUCTION

There is convincing evidence that excess body weight is associated with increased risk for late onset (> 50 years of age) colorectal cancer (CRC) [1, 2]. Interestingly, sex and hormonal status appear to play a role in this relationship [3]. Several epidemiological studies have demonstrated that obesity increases the risk of and mortality from CRC in males [4–6]. However, the relationship in females is somewhat inconsistent, likely at least in part due to a potential protective effect that reproductive hormones may have on CRC risk. For instance, evidence suggests that postmenopausal women have an increased risk of CRC but this effect may be limited to individuals not currently using hormone replacement therapy (HRT) [7–10]. Taken together, the current literature suggests that 1) sex disparities are evident in obesity-enhanced CRC, and 2) hormonal status likely plays a role in CRC risk in women. Despite these findings, controlled animal studies to substantiate these claims and to determine potential mechanisms are lacking.

Recent advances in body composition assessment, including computerized tomography (CT), have indicated that distribution of fat may be a risk factor for CRC and a potential mechanism for the observed sex-driven differences [11–14]. In a cohort of stage I-III CRC patients, abdominal adipose tissue quantity and distribution were prognostic of all-cause mortality and the shapes of these associations were modified by patient sex [15]. Further, in a recent study using CT, colorectal adenoma was significantly associated with visceral adipose tissue in men but not women [11]. Similarly, in patients undergoing first-line bevacizumab-based treatment for metastatic CRC and in patients that received adjuvant chemotherapy, visceral adipose tissue independently predicted poorer outcomes [16, 17]. It is important to note that men preferentially store adipose tissue viscerally whereas women more likely store it subcutaneously [18]. This is likely to be driven by sex hormones given their ability to influence adipose tissue deposition [19]. Indeed it is well known that sex steroids are important regulators of both adipocyte development and function, although molecular details of their actions have not been completely unearthed [20, 21]. The literature suggests that estrogen is a negative regulator of fat mass *in vivo* [20, 21]. For example, the decline in estrogen in postmenopausal women is associated with adipose tissue accretion [22]. Further, a recent study using a mouse model that can track adipogenesis *in vivo* showed that estrogen is one of the factors that contribute to the sex dependent depot-differences in adipocyte development [23]. Interestingly, androgens are known to suppress adipogenesis in men and preferential reduction in visceral depots by androgen treatment in men has been documented [24]. Thus, the influence of sex and hormonal status on adipose tissue accumulation may contribute to the reported increased incidence of CRC in males and the protective effect of HRT in postmenopausal females.

Extensive epidemiological, clinical, and preclinical literature acknowledges that adipose tissue provokes metabolic derangements including inflammatory processes, insulin resistance, and altered adipokine profile. These perturbations have been associated with CRC risk and are likely biological factors that link obesity to CRC risk [25]. For instance, the homeostasis model of risk assessment-insulin resistance (HOMA-IR) has been reported to be associated with risk for CRC [26]. Further, a meta-analysis documented a positive relationship between insulin therapy and increased risk of CRC in patients with type 2 diabetes [27]. Interestingly, the aforementioned factors are influenced by sex and hormone status. For instance, obese men, compared to obese women, have lower insulin sensitivity and elevated glucose levels promoting insulin resistance [28]. In rodent models, male mice exhibit greater infiltration of pro-inflammatory macrophages in adipose tissue compared to females but this protection appears to be lost in female mice that are ovariectomized [29]. Despite the postulation that these metabolic derangements link obesity to CRC risk, there has been little experimental investigation to solidify this presumption. This has undoubtedly been hindered by the lack of appropriate animal models to study obesity-enhanced CRC.

In order to investigate sex disparities in obesity-enhanced CRC, we utilized the subcutaneous MC38 tumor model, which allowed us to establish an obese phenotype with associated metabolic dysfunction prior to the initiation of cancer. As hormone status is an influential risk factor for both obesity and CRC, we also sought to examine the effect of ovarian hormone deficiency on obesity-enhanced CRC. Finally, we examined body composition and metabolic derangements including inflammatory processes, insulin resistance, and adipokines as potential contributing factors to obesity-enhanced CRC. Overall, this study provides insight into the contributions of hormonal status, fat distribution (i.e., visceral versus subcutaneous adipose tissue), and local versus systemic effects of obesity (metabolism and inflammation) on CRC risk.

## RESULTS

### OVX mice presented with greatest adiposity, specifically subcutaneous adiposity, following 20–21 weeks of HFD consumption

**Figure 1:**
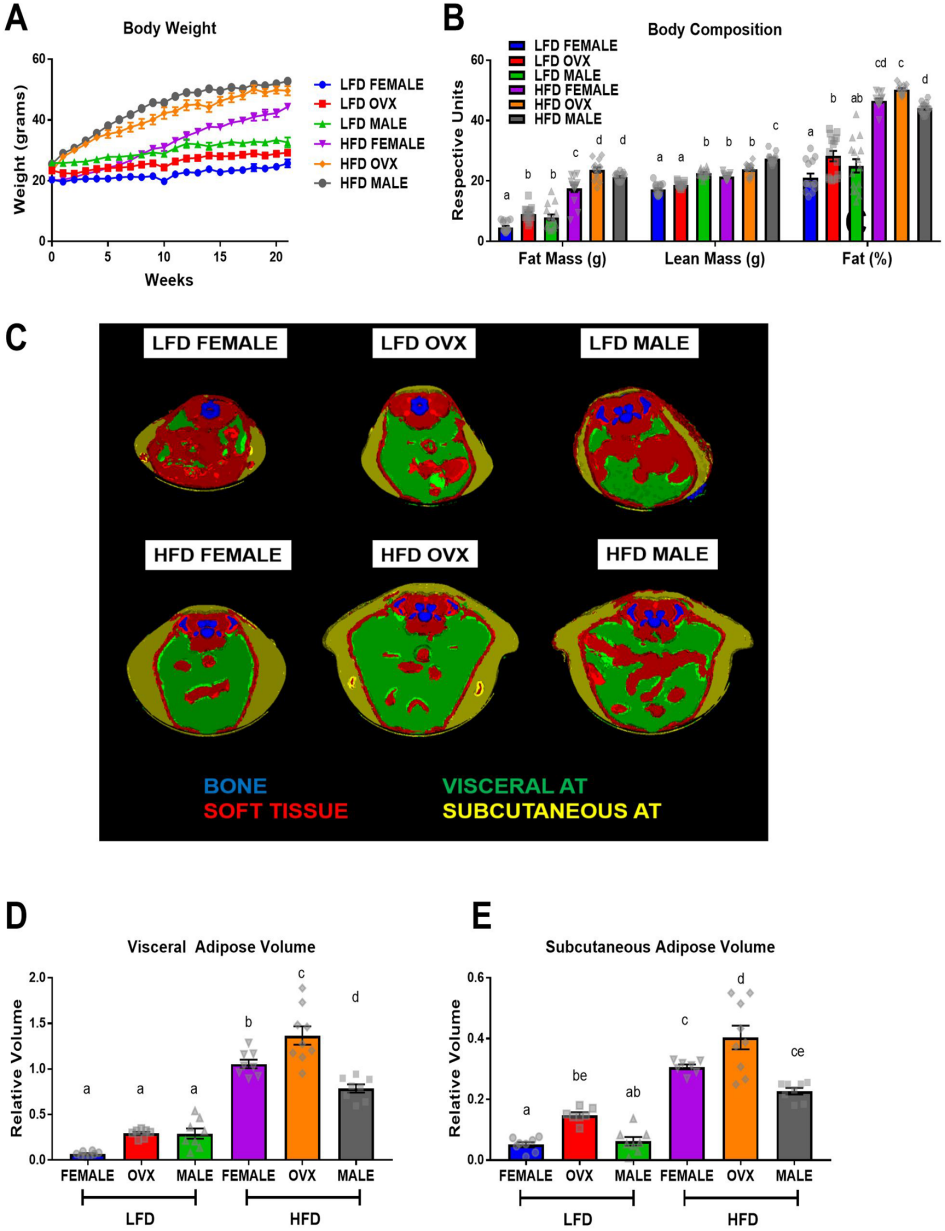
Sex disparities in body weight, body composition and adipose tissue distribution after dietary treatment. (**A**) Mouse body weight throughout the diet feeding, (**B**) body composition, fat mass, lean mass, and fat%, respectively via DEXA (**C**) Representative microCT images of defined objects of bone, soft tissue, visceral fat, subcutaneous fat, based on density thresholds (**D** and **E**) Quantification of adipose volume based on defined objects within microCT scans. Volumes relative to soft tissue volumes. Values are mean ± SE; *n* = 8–15 mice per group. Bar graphs not sharing a common letter are significantly different from one another (*p* ≤ 0.05).

At 9 weeks of age mice received either a sham or ovariectomy surgery to establish male, female, or OVX groups. Two weeks following surgery, mice were fed a 40% high-fat diet (HFD) or a purified low-fat diet (LFD) for 20–21 weeks. An obese phenotype was successfully achieved as the HFD-fed mice gained significantly more body weight (Figure 1A, *p* < 0.05) and fat mass (Figure 1B, *p* < 0.01), and displayed a significantly greater body fat % (Figure 1B, *p* < 0.01) compared to LFD fed mice. Within HFD, male mice and OVX mice exhibited increased fat mass compared to female mice (Figure 1B, *p* < 0.05). Male HFD-fed mice had greater lean mass compared to female and OVX mice fed the same diet (Figure 1B, *p* < 0.01). OVX mice exhibited significantly increased body fat % (Figure 1B, *p* < 0.05) compared to male mice although there was no significant difference between female and OVX within the HFD group nor between HFD female and HFD male. A separate group of mice was used for microCT analysis and revealed differences in the distribution of body fat following HFD feedings (Figure 1C–1E, *P* < 0.05); visceral and subcutaneous adiposity is unique within obese male, female, and OVX mice. HFD OVX mice presented with the largest adipose volumes in both visceral (Figure 1D) and subcutaneous depots (Figure 1E). Compared to HFD male and female mice, OVX mice within the same diet had significantly increased visceral and subcutaneous adipose volumes (Figure 1D and 1E, *p* < 0.001). HFD-fed female mice had increased visceral adipose volume (Figure 1D, *p* < 0.05) but not subcutaneous adipose volume (Figure 1E, *p* = 0.10) compared to HFD-fed male mice. These data confirm that our HFD feeding regime results in increased adiposity. Specifically, OVX mice fed a HFD presented with greatest adiposity, particularly subcutaneous adiposity, following 20–21 weeks of HFD consumption. After confirming increased adiposity in HFD fed mice we next wanted to assess metabolic markers.

### Female mice exhibit protection against high-fat diet induced insulin resistance

**Figure 2:**
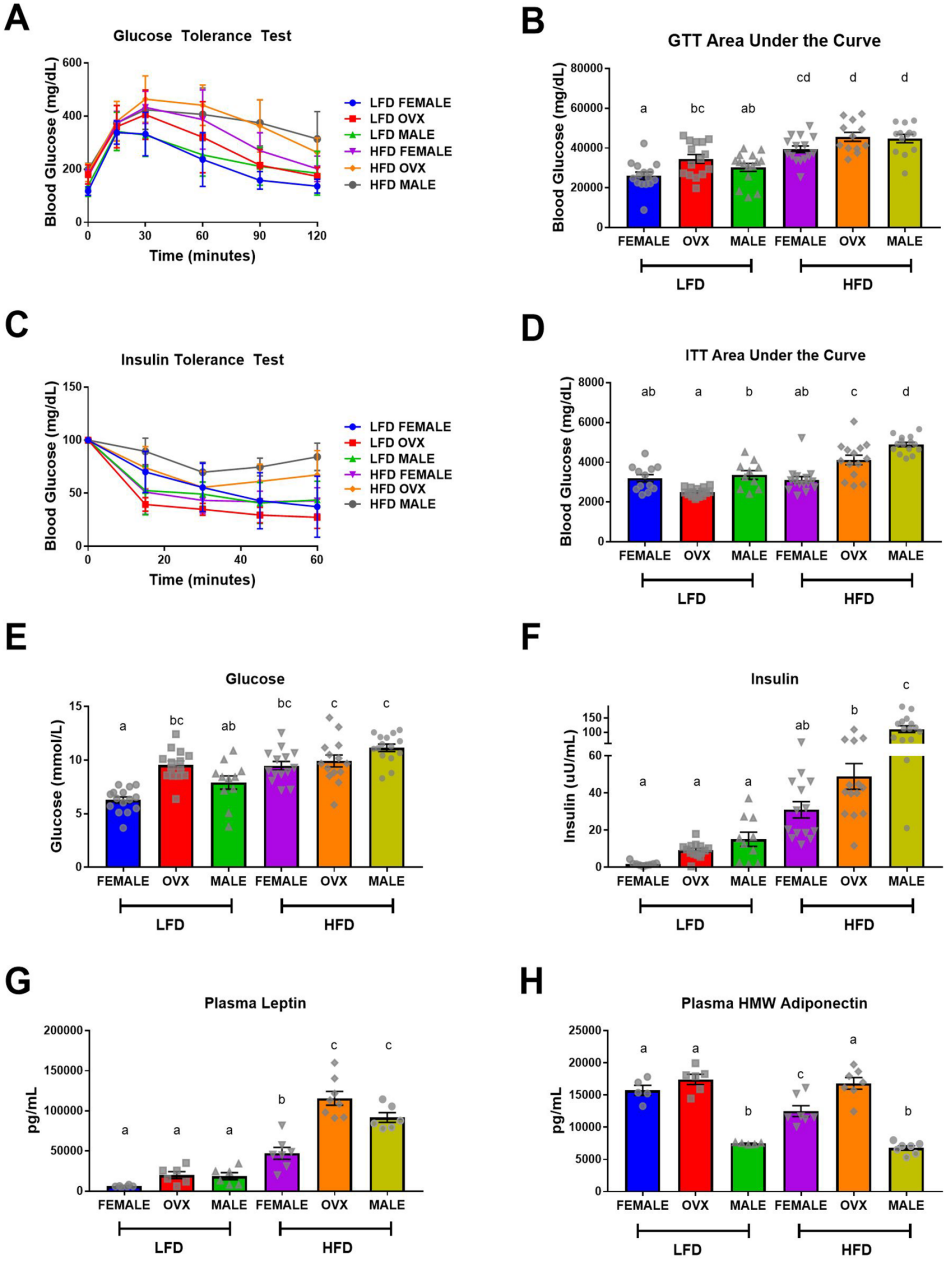
Impaired metabolic function following high-fat diet feeding. (**A** and **B**) Intraperitoneal glucose tolerance test (GTT) and corresponding area under the curve (AUC), (**C** and **D**) Intraperitoneal insulin tolerance test (ITT) following a 5-hour fast and corresponding AUC. (**E**) Fasting (5 hour) blood glucose levels, (**F**) Fasting (5 hour) plasma insulin levels, (**G** and **H**) ELISA quantification of fasting plasma adipokines, leptin and high molecular weight adiponectin, respectively. Values are mean ± SE; *n* = 8–15 mice per group. Bar graphs not sharing a common letter are significantly different from one another (*p* ≤ 0.05).

HFD-fed mice displayed impaired glucose tolerance and insulin action (Figure 2A–2D) and hyperglycemia and hyperinsulinemia (Figure 2E and 2F) compared to LFD-fed mice (*p* < 0.001). Taken together these data indicate that 20–21 weeks of HFD feeding results in metabolic dysfunction consistent with insulin resistance. Within the HFD group, male mice exhibited the most severe insulin resistance (Figure 2D and 2F, *p* < 0.001), suggesting that female mice may exhibit some protection against obesity-associated insulin resistance. However, this observed protection was not as prevalent in the HFD OVX group, which presented with worsened insulin action (Figure 2D and 2F, *p* < 0.001), compared to HFD females. Within the LFD group, OVX mice presented with slightly increased glucose tolerance test (GTT) area under the curve (AUC) and fasting blood glucose levels compared to female mice (Figure 2B and 2E, *p* = 0.04 and *p* < 0.001 respectively). Additionally, LFD males presented with increased insulin tolerance test (ITT) AUC compared to LFD OVX mice (Figure 2D, *p* = 0.01). Assessment of circulating adipokines, revealed a main effect of diet with increased leptin (Figure 2G, *p* < 0.01) and decreased high molecular weight (HMW) adiponectin (Figure 2H, *p* = 0.02) for HFD mice compared to LFD mice. HFD-fed OVX mice had the highest levels of leptin, which was significantly different from female (Figure 2G, *p* < 0.001) but not male mice fed the same diet. Interestingly within HFD, HMW adiponectin was decreased in female and male mice compared to OVX (Figure 2H, *P* < 0.05) with a further decrease in male mice when compared to female mice (Figure 2H, *P* < 0.05). Within LFD mice, there was no difference in circulating leptin levels between male, female, and OVX mice (Figure 2G); however, male mice had significantly decreased HMW adiponectin compared to female and OVX mice (Figure 2H, *p* < 0.001). These data confirm the metabolic perturbations associated with HFD feedings and the protective role of female hormones in this response. We next wanted to determine whether the established adiposity and metabolic outcomes were linked to increased tumorigenesis.

### MC38 subcutaneous tumor growth accelerated in HFD OVX mice

**Figure 3:**
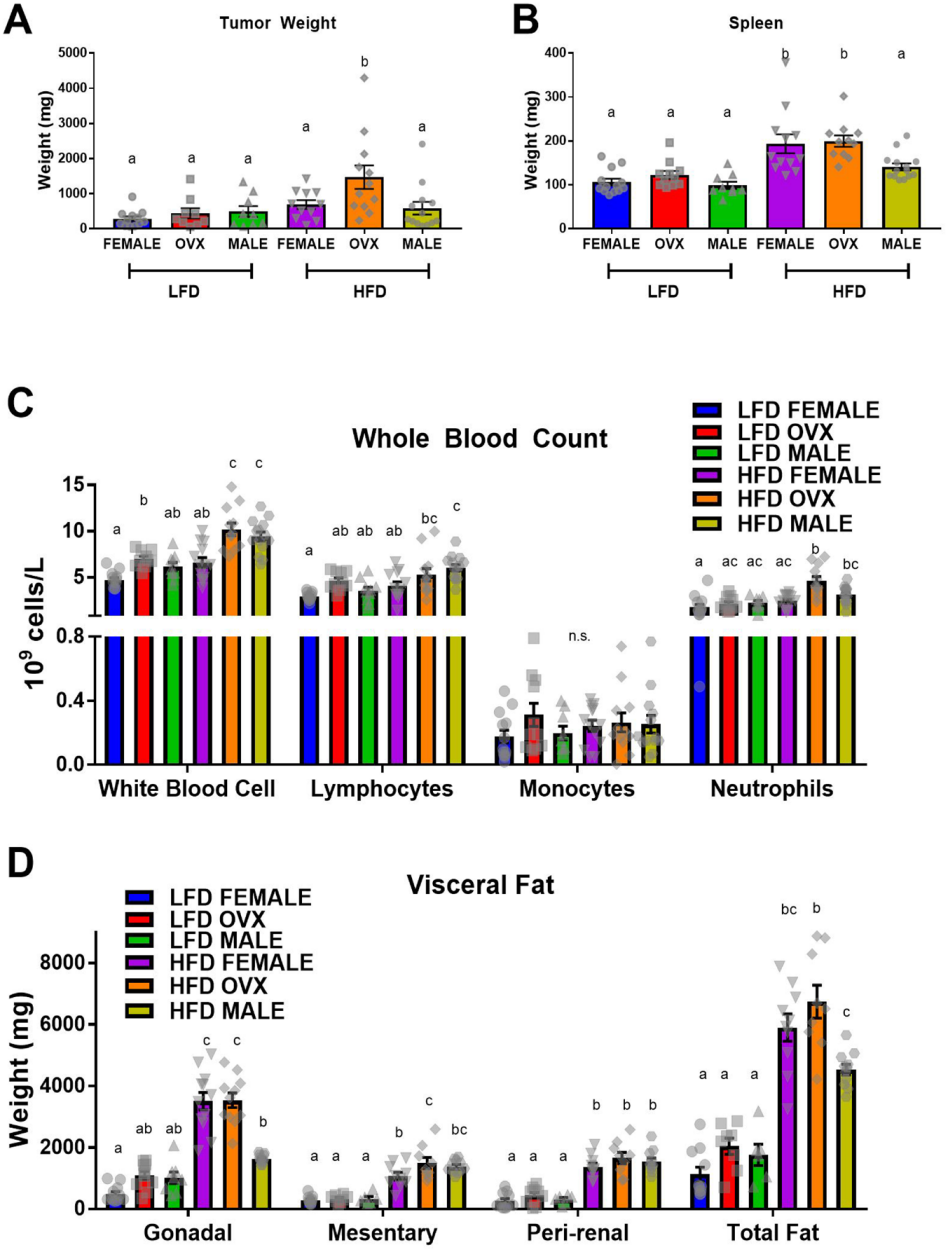
High-fat diet feeding increased MC38 tumor weight (**A**) Tumor weight (**B**) Spleen weight. (**C**) Complete blood count of immune cells in whole blood. (**D**) Weight of visceral adipose depots; total fat equals the sum of gonadal, mesentery and peri-renal weights. Values are mean ± SE; *n* = 8–15 mice per group. Bar graphs not sharing a common letter are significantly different from one another (*p* ≤ 0.05).

Following MC38 cell injection, tumors were allowed to grow for 3 weeks before the mice were euthanized and tumor weights were measured. The tumor cells engrafted and expanded in all mice. Consistent with epidemiological evidence, main effects revealed that HFD resulted in increased tumor weight compared to LFD (Figure 3A, *p* < 0.01). This was associated with increased spleen weight (Figure 3B, *p* < 0.01) and numbers of circulating white blood cells, specifically lymphocytes and neutrophils, in the HFD mice compared to LFD (Figure 3C, *p* < 0.01). As expected, HFD mice maintained increased adiposity of visceral depots following the 23–24 week feeding period compared to LFD mice (Figure 3D; *P* < 0.05). As expected, there was a positive correlation between total fat and tumor size (r^2^ = 0.294, *p* = 0.0008). Interestingly, the HFD-fed OVX group presented with the largest tumors of any group; significantly larger compared to female (*p* = 0.04) or male (*p* < 0.01) groups within the same diet treatment (Figure 3A). This increase in tumor weight within HFD OVX was associated with increased circulating neutrophils compared to female (Figure 3C, *p* < 0.01) but not male mice within the same diet. However, contrary to the epidemiological evidence, HFD feeding in male mice did not significantly increase tumor weight compared to LFD male mice (Figure 3A). Further, the tumor onset resulted in reduced gonadal adipose tissue weights measured 3 weeks following tumor initiation compared to female or OVX mice within HFD and reduced total adipose tissue compared to OVX mice within HFD (Figure 3D, *p* < 0.01). Despite the reduced tumor weight and gonadal adipose tissue, HFD male mice presented with increased white blood cells compared to female mice of the same diet (Figure 3C, *p* < 0.01), similar to the HFD OVX mice. Within the LFD group, there was no significant difference between tumor weight, spleen weight, neutrophil count, or total visceral fat between male, female and OVX mice. Lastly, there was no metastasis documented in any of the mice nor was there any mortality. These data confirm that HFD feedings can lead to an increase in tumorigenesis (main effect), which appears to be largely due to the increase in tumorigenesis in the OVX mice. We next wanted to assess whether adipose tissue inflammation, insulin-like growth factor 1, or TAMs were associated with this response.

### Adipose tissue inflammation is exacerbated in HFD at the time of tumor initiation

**Figure 4:**
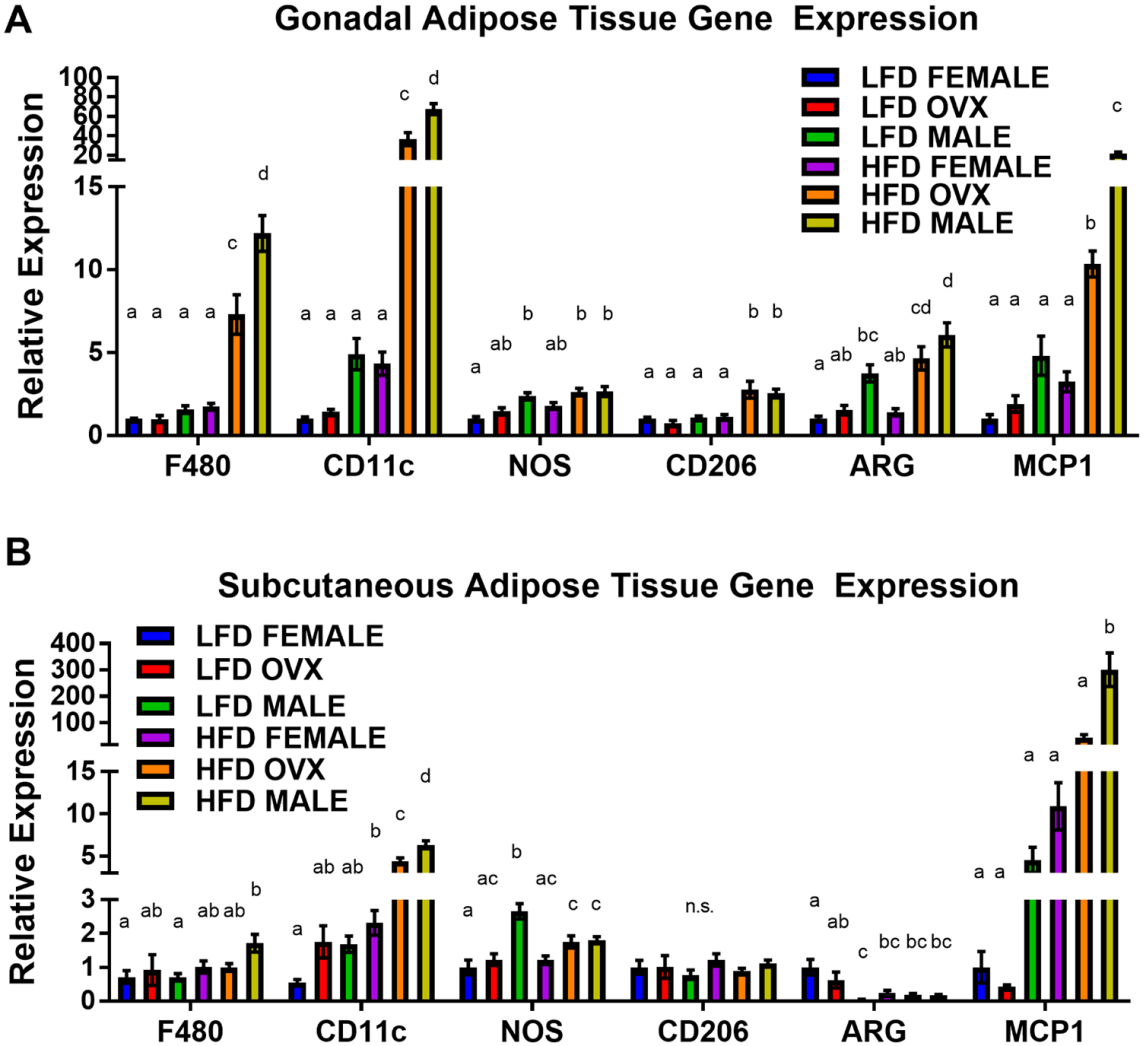
Increased pro-inflammatory macrophages within obese adipose tissue depots. (**A**) Relative gene expression of F480, CD11c, NOS, CD206, ARG, and MCP1 of mRNA isolated from gonadal (epididymal/perimetrial) adipose tissue. (**B**) Relative gene expression mRNA isolated from subcutaneous adipose tissue. Ct values relative to average of multiple internal controls determined using qBASE pro software analysis. Ct values normalized to LFD Female group. Values are means ± SE; *n* = 8–9 mice per group. Bar graphs not sharing a common letter are significantly different from one another (*p* ≤ 0.05).

Following 20–21 weeks of diet treatment, baseline analysis of adipose tissue revealed a main effect of HFD on adipose tissue macrophage markers, suggesting increased macrophage-associated adipose tissue inflammation at the time of tumor injection. As expected, a main effect of HFD was achieved through increased F4/80, CD11c and monocyte chemoattractant protein 1 (MCP1) gene expression compared to LFD within both the gonadal (Figure 4A, *p* < 0.01 for all) and subcutaneous (Figure 4B, *p* = 0.01 *p* < 0.01 and *p* < 0.01, respectively) adipose tissue depots. Within the HFD group, male mice presented with the most severe macrophage associated gonadal adipose tissue markers with significantly increased F4/80, CD11c, CD206, arginine (ARG), and MCP1 compared to female mice (Figure 4A, *P* < 0.01). HFD OVX mice presented with similarly increased macrophage associated gonadal tissue markers (F4/80, CD11c, CD206, ARG and MCP1) compared to female mice of the same diet (Figure 4A, *p* < 0.01), although these adipose tissue markers were not as elevated as that in the males. Among the LFD treated mice, there was no significant difference in gonadal F4/80, CD11c, CD206, or MCP1 gene expression between male, female, or OVX mice. However, NOS and ARG gene expression were significantly increased in LFD male mice when compared to LFD female mice.

Within the subcutaneous adipose tissue depot macrophage-associated inflammation was not as severe as the visceral adipose tissue; however, several unique differences were revealed. Although overall macrophage expression F4/80 was not significantly increased (*p* = 0.16), subcutaneous adipose tissue of HFD-fed male mice exhibited increased CD11c (*p* < 0.01) and MCP1 (*p* < 0.01) gene expression compared to HFD female mice (Figure 4B). This increase in pro-inflammatory macrophage gene expression, CD11c, also was present in the OVX mice compared to female mice within HFD (*p* < 0.01). In subcutaneous adipose tissue, increased gene expression of NOS and decreased gene expression of ARG were observed in LFD male when compared to LFD female mice (Figure 4B, *p* < 0.05). No significant changes were observed in the gene expression of F4/80, CD11c, CD206, and MCP1 within the LFD groups. Consistent with the metabolic function data, intact female mice are, to a certain extent, protected against obesity-induced adipose tissue inflammation while OVX mice have similar levels of inflammation as male mice. Given its association with tumorigenesis, we next evaluated insulin-like growth factor-1 (IGF-1) in the local environment.

### Localized subcutaneous adipose tissue of HFD OVX mice released insulin-like growth factor 1 which may have accelerated tumor growth

**Figure 5:**
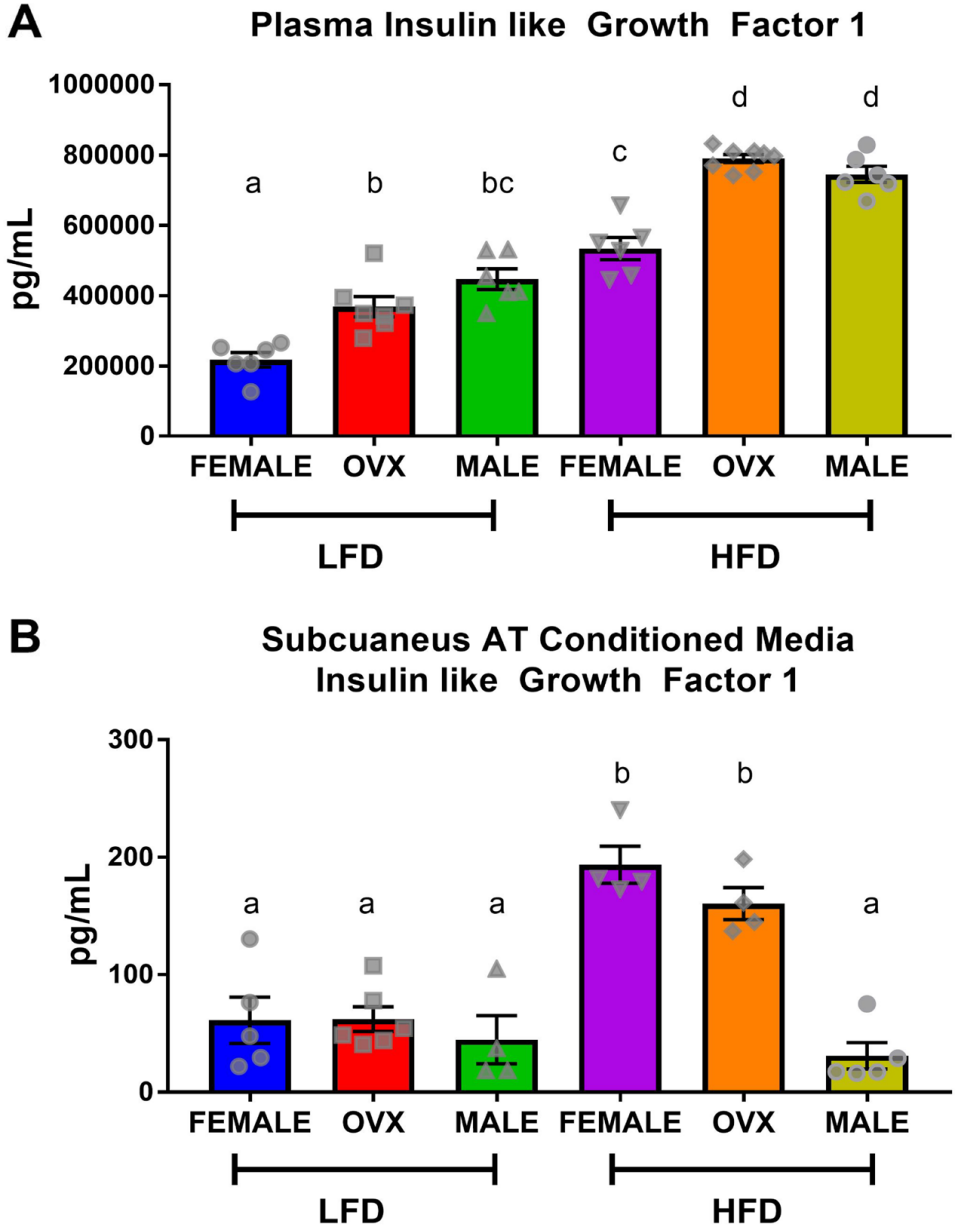
Circulating insulin-like growth factor-1 is not consistent with localized subcutaneous adipose tissue IGF. (**A**) ELISA quantification of plasma IGF levels following 5 hour fast. (**B**) ELISA quantification of IGF within subcutaneous adipose tissue conditioned media from the dorsal-lumbar depot. Values are means ± SE; *n* = 8–9 mice per group. Bar graphs not sharing a common letter are significantly different from one another (*p* ≤ 0.05).

Following 20–21 weeks of diet treatment, plasma and conditioned media collected from subcutaneous adipose tissue explants contained significantly higher levels of IGF-1 in HFD mice compared to LFD mice (Figure 5A and 5B). Within HFD, circulating IGF-1 levels trended similarly to circulating insulin levels (Figure 2F), with HFD-fed OVX and male mice exhibiting increased plasma IGF-1 compared to female mice (Figure 5A, *p* < 0.01). Although contrary to circulating insulin, there was no significant difference between HFD OVX and male circulating IGF-1 levels. Analysis of subcutaneous adipose tissue conditioned media revealed that despite increased plasma IGF-1, HFD male mice had significantly decreased localized subcutaneous IGF-1 compared to HFD female or HFD OVX mice (Figure 5B, *p* < 0.01). This suggests that subcutaneous adipose tissue of HFD female and OVX mice release greater amounts of IGF-1 than HFD male adipose tissue. Finally, we assessed TAMs as a potential mechanism for the increased tumorigenesis in OVX mice.

### M2-like tumor-associated macrophages increased in OVX tumors compared to female tumors

**Figure 6:**
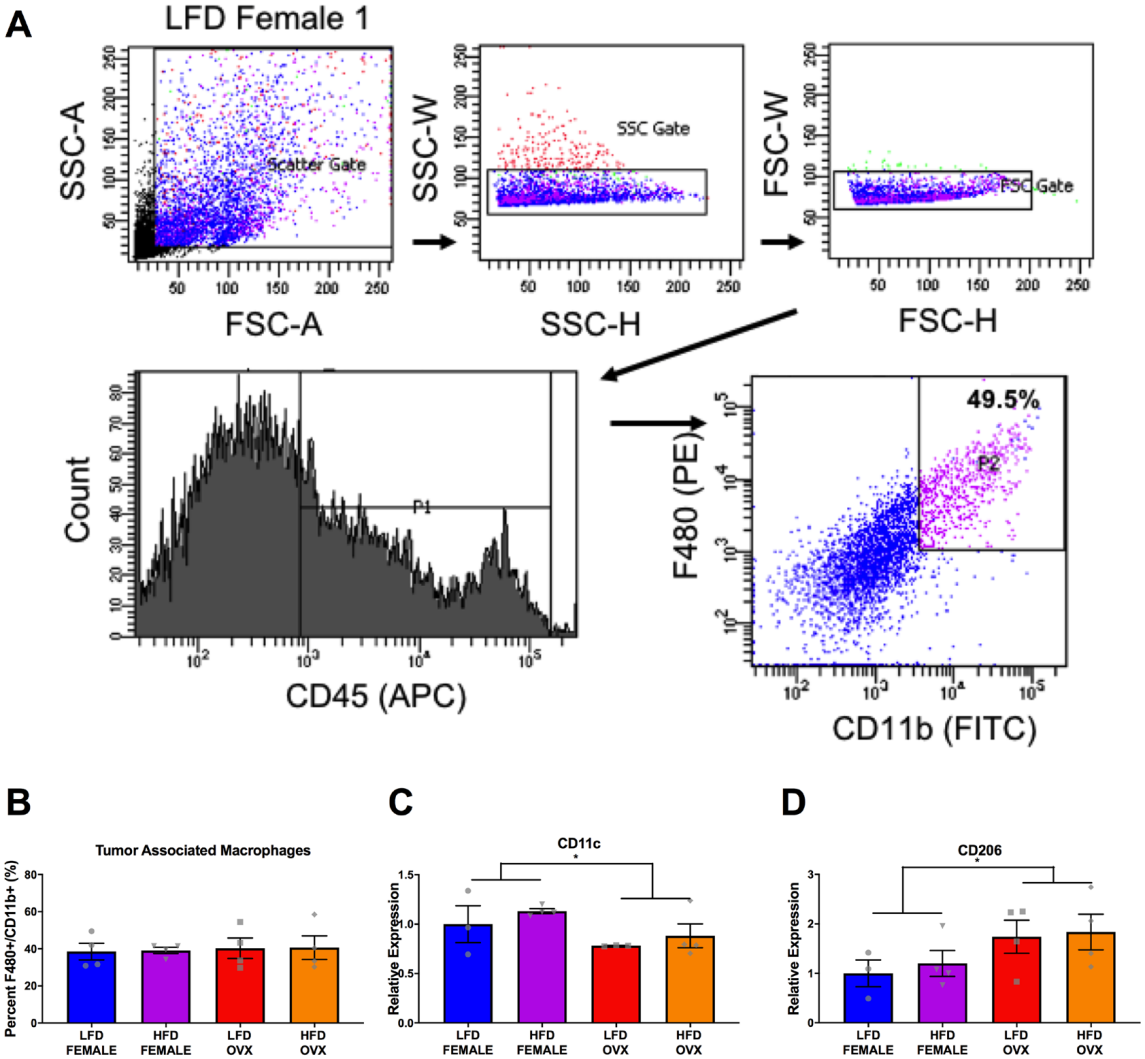
Differential tumor-associated macrophage phenotype in female and OVX mice. (**A**) Representative gating strategy for sorting of tumor associated macrophages, defined as CD45+ F4/80+ CD11b+ cells. (**B**) Percentage of tumor associated macrophages within tumors at the time of sort. (**C** and **D**) Relative gene expression of CD11c (C) and CD206 (D) in collected tumor associated macrophages. Ct values relative to average of multiple internal controls determined using qBASE pro software analysis. Ct values normalized to LFD Female group. Values are means ± SE; *n* = 3–4 mice per group. Bar graphs not sharing a common letter are significantly different from one another (*p* ≤ 0.05).

Due to the unchanged tumor growth observed in the LFD compared to HFD male mice (Figure 3A), we focused solely on the interaction between female and OVX mice within respective diets. Following tumor excision, tumor associated macrophages (TAMs) were defined as F4/80 and CD11b double positive cells from a CD45 positive gate and were collected using fluorescence activated cell sorting (FACS) (Figure 6A). Although there were differences in tumor weight (Figure 3A) between HFD OVX and female mice, there were no significant differences in the percentage of TAMs within these tumors (Figure 6B). However, upon further investigation of the gene expression in these isolated TAMs, CD11c, an M1 macrophage marker was significantly decreased in OVX mice (Figure 6C, *p* = 0.05), consistent with a significant increase in expression of CD206, an M2-macrophage marker (Figure 6D, *p* = 0.02) compared to female mice independent of diet. This indicates that irrespective of tumor size or diet, OVX mice possess more pro-tumor macrophages within the tumor microenvironment compared to female mice.

## DISCUSSION

There is epidemiological evidence for sex disparities in obesity-enhanced CRC that may be driven by sex hormones. We used the subcutaneous MC38 tumor mouse model and diet-induced obesity to examine sex-differences in obesity-enhanced CRC. Further, ovariectomy was utilized to assess the contribution of ovarian hormones to this response. Initially, we characterized sex-specific phenotypes of obesity following 20–21 weeks of high-fat diet feeding. Despite increased adiposity, female mice exhibited protection against obesity-associated insulin resistance compared to male mice, although this protection was diminished in OVX mice. We observed enhanced tumor growth following diet-induced obesity (main effect of HFD) with post-hoc analysis revealing the largest increase seen in OVX mice. However, male mice failed to coincide with epidemiological data and did not exhibit increased tumor growth following high-fat diet feeding, despite severe insulin resistance. We believe that these unique sex differences in obesity enhanced tumor growth may be driven by the subcutaneous microenvironment at the initial time point of cancer cell injection, and propose potential mechanisms relating to OVX specific tumor growth within a high-fat-diet. First, we observed that prior to the injection of tumor cells at 20–21 weeks of high-fat-diet feeding, OVX mice had significantly increased visceral and subcutaneous fat compared to male and female mice (Figure 1C–1E). Furthermore, the subcutaneous adipose tissue depots within HFD OVX exhibited more severe macrophage-associated inflammation compared to female, but not male mice (Figure 4B). Second, the conditioned media collected from subcutaneous adipose tissue of HFD OVX, contained significantly higher IGF-1 levels compared to male, but not female mice (Figure 5B). Third, OVX TAMs had increased M2-like gene expression compared to female mice. Taken together these data suggest that accelerated subcutaneous tumor growth within obese OVX mice is associated with an increase in subcutaneous adiposity and adipose inflammation, an IGF-1 rich subcutaneous microenvironment, and an increased presence of M2-like TAMs.

The association between obesity and CRC has been suggested in both epidemiological and *in vivo* mouse studies. Epidemiological evidence has further uncovered a more significant relationship in men than in women [4–6]; sex hormones have been implicated in this sex-driven difference. For example, a meta-analysis of 18 observational studies showed a 20% reduction in colon cancer incidence among women who had ever used HRT [9]. Further support for this relationship comes from *in vitro* findings that have shown that estradiol in addition to estrogen receptor β agonist can inhibit proliferation and migration of MC38 cells [30, 31]. In several *in vivo* studies, exposure of ovariectomized rats to estrogen reduced the rate of colon tumors by 71% [32, 33]. In addition, in the Apc^Min/+^ mice, ovariectomy resulted in an increased number of polyps, whereas estrogen replacement reduced the number of polyps to baseline levels [34]. Our data is consistent with these findings, as the HFD OVX mice presented with larger tumors than HFD female mice. Yakar *et al.*, revealed similar increases in tumor growth of obese OVX mice compared to female mice in the same model [35]. Contrary to our findings, this group reported obesity-enhanced tumor growth in males in addition to female and OVX mice [36]. However, it should be noted that this group did not directly compare all 3 groups (male, female and OVX) within the same experiment. The length of high-fat diet feeding (10 wks versus 20–21 wks) and the composition of fat in the diets (35% versus 40%) may further explain the disparate findings. One limitation of our study is that we did not include an estrogen replacement group to determine if the effects of OVX are due to estrogen alone nor did we include a male group that was administered estrogen; additional work is required to establish whether the documented effects are mediated by estrogen.

Although there is evidence to support sex and hormonal influences on obesity enhanced CRC, a definite mechanism has yet to be elucidated. Epidemiological evidence suggests that distribution of adiposity may play an important role in CRC [37]; abdominal adiposity is positively correlated with CRC, thus supporting clinical data correlating obese males with increased risk of CRC [38, 39]. Utilizing highly accurate micro CT analysis, we are the first to characterize differences in adipose distribution with lean and obese male, female, and OVX mice. As we used an ectopic colorectal tumor model, we rationalized that subcutaneous adipose tissue is the localized adipose depot relative to this particular model. Given the increased tumorigenesis in obese mice, our data suggests that increased subcutaneous adiposity may accelerate subcutaneous tumor growth. OVX mice, the group with the greatest tumor size, had the largest increase in subcutaneous adiposity, providing further evidence for a link between local adiposity and enhanced tumorigenesis. It is important to point out that the use of an ectopic model is limiting; typically, CRC will develop within the colon or rectum and not in an environment rich in subcutaneous adipose tissue. Thus, the translational relevance of the study is limited. Indeed, it has been documented that the mechanical and biochemical properties of the adipose tissue can promote malignancy – at least in breast cancer [40–42]. Follow up studies using orthotopic models are critical to confirm findings and for translation to human patients.

Adipose tissue macrophage associated inflammation has been a proposed driving force for the link between obesity and CRC [43]. We observed that prior to tumor onset, HFD OVX mice presented with elevated expression of CD11c (M1 pro-inflammatory macrophage marker) and MCP-1 (macrophage chemoattractant) within adipose tissue depots, specifically the subcutaneous adipose tissue, compared to female mice. This is consistent with previous unpublished data from our group showing that OVX leads to an increase in adipose tissue inflammation following high-fat diet feedings, which is reversed by estrogen replacement (unpublished). Interestingly, macrophages can release inflammatory cytokines such as tumor necrosis factor α (TNF-α), which can activate the inflammatory pathways NFκB and STAT3 that mediate proliferation, invasion, angiogenesis, survival and metastasis [44, 45]. The increased presence of pro-inflammatory adipose tissue macrophages in the subcutaneous adipose tissue depot of HFD OVX mice may have contributed to the accelerated tumor growth that was observed 3 weeks post MC38 injection. One caveat to this is that HFD male mice also exhibited elevated levels of M1 macrophage expression and MCP-1, and even significantly higher than OVX HFD mice, yet did not display enhanced tumorigenesis. Secondly, literature supports an increase in aromatase, the rate-limiting enzyme for estrogen synthesis, in adipose tissue from obese post-menopausal women [46]. If estrogen is playing a role in adipose tissue inflammation this would be expected to improve the inflammatory profile of adipose tissue, yet it has been associated with increased breast tumorigenesis [46]. This highlights the complexity of the relationship between obesity and cancer as well as the need for more work in this area. It is also important to note that the analysis of adipose tissue inflammation was performed on whole adipose tissue using qPCR as opposed to FACS precluding definite conclusions on the source of these markers; thus, while it is clear that these markers are present in adipose tissue we cannot rule out that cells other than macrophages are contributing to this response.

Evidence suggests that obesity promotes CRC by activating the insulin/IGF-1 signaling pathway [47–49]. Insulin and IGF-1 are leading determinants of proliferation and apoptosis and therefore likely to influence colon cancer growth [50, 51] and tumorigenesis [52]. Studies in animal models have shown that manipulation of insulin and IGF-1 levels may significantly affect CRC [52]. Our findings provide new evidence showing that IGF-1 is increased in conditioned media taken from subcutaneous adipose tissue of HFD female and OVX mice but not in HFD male mice (Figure 5B), providing a potential explanation for the tumorigenesis findings. The disparity between tumor growth in HFD female and OVX cannot be explained by IGF-1 as there was no significant difference in adipose tissue IGF-1 levels between HFD female and OVX mice. While male mice failed to show an increase in subcutaneous adipose tissue IGF-1, they did show a significant increase in circulating insulin and plasma IGF-1 levels that were similar to those observed in OVX mice. Others have shown that insulin can increase colon tumor growth. For example, Hvid *et al.* reported that treatment with insulin accelerated tumor growth, specifically in the MC38 subcutaneous tumor model [53]. However, we do not believe that insulin and circulating IGF-1 are contributing to tumorigenesis in our study given the failure to see an increase in CRC in HFD male mice. These results suggest that increased local IGF-1 release may have enhanced MC38 proliferation in our tumor model. However, additional studies are necessary to confirm this hypothesis including the assessment of tumor IGF-1, which was not performed in the current study.

Macrophages have emerged as important mediators of pro-tumoral processes. TAMs are conditioned by the tumor microenvironment and exert pro-tumoral functions. Factors such as transforming growth factor β (TGF-β) and interleukin 10 (IL-10) have the potential to modulate and polarize monocytes mainly into M2 macrophages that can then promote proliferation, invasion and metastasis, angiogenesis, and matrix deposition and remodeling [54]. The majority of studies have reported that high levels of TAMs are associated with poor prognosis in CRC [55, 56]. Further, there is some evidence that estrogen can affect the polarization of macrophages in the tumor microenvironment, albeit not in a CRC model [57]. Therefore, in an attempt to explain the increased tumor growth in HFD OVX mice compared to HFD female mice we examined expression of M1 (anti-tumor) and M2 (pro-tumor) markers in TAMs. The TAMs within OVX tumors had higher expression of CD206 (M2 marker) with reduced expression of CD11c (M1 marker) compared to female mice. This shift in the M1/M2 like TAM ratio suggests a more tumoricidal environment that coincides with the increased tumor size in HFD OVX mice. These results indicate a potential for ovarian hormones to influence TAM phenotype. It is important to note that TAMs were isolated from tumors of different sizes; therefore, it is unclear whether this altered macrophage phenotype directly impacted tumor growth or is simply a consequence of the difference in tumor size.

Overall, our findings regarding a role of ovarian hormones in obesity-enhanced tumorigenesis are consistent with the epidemiological literature implicating HRT as protective against CRC risk in postmenopausal females. The link between obesity and CRC is undoubtedly complex and likely to involve several interrelated mechanisms as explored in this study. We provide some clear candidate mechanisms that are likely responsible for the enhanced tumor growth in HFD OVX mice. What is less clear is the failure to detect a significant increase in tumorigenesis in HFD male mice, which is inconsistent with the epidemiological literature. This finding is surprising given that these mice exhibited increased adiposity, insulin resistance, and subcutaneous adipose tissue inflammation – all of which have been linked to CRC. It is possible that IGF-1, or lack thereof, released from the subcutaneous tissue of HFD male mice may explain these findings in this model. Further studies using IGF-1 manipulation techniques would be needed to test this hypothesis.

In conclusion, our study confirms that established obesity following 20–21 weeks of high-fat-diet feedings enhanced the growth of subcutaneously implanted MC38 tumors, but this appears to be largely due to the increase in tumorigenesis in the OVX mice. We established that diet induced obesity and associated insulin resistance manifest differently in male, female, and OVX mice. Although we were unable to completely replicate epidemiological data, we report that obese OVX mice are most vulnerable to accelerated subcutaneous tumor growth in the MC38 model. Our data suggest several potential mechanisms including increased local adiposity, increased local inflammation and IGF-1, and polarization of macrophages to an M2 phenotype, providing insight into this obesity enhanced tumor growth in OVX mice.

## MATERIALS AND METHODS

### Animals

Male and female wildtype (WT) *C57BL/6* mice were purchased from the Jackson Laboratories (Bar Harbor, ME) and cared for at the Department of Laboratory Animal Resources at the University of South Carolina. Mice (*n* = 23–24/group) were housed 5 per cage, maintained on a 12:12-h light-dark cycle in a low stress environment (22°C, 50% humidity, low noise) and given food and water *ad libitum*. Principles of laboratory animal care were followed, and the Institutional Animal Care and Usage Committee of the University of South Carolina approved all experiments. At 9 weeks of age, all male mice underwent a sham surgery and all female mice underwent either a sham or ovariectomy (OVX) surgery. Briefly, mice underwent anaesthesia maintained with 2% isoflurane and oxygen before a dorsal incision was made to the skin then the muscle layers. Both uterine horns were tied with non-absorbable suture 5–0 (cat # S-G518R13) and ovaries were removed. In the case of the sham-operated mice, the ovaries were exteriorized and then placed back intact into the abdominal cavity. The muscle wall was sutured with 5–0 absorbable suture (cat # S-G518R13-U) and wound clips were used to close the skin incision. Buprenorphine was given as a pain reliever subcutaneously at a dose of 0.043 mg/kg. Wound clips were removed one week after surgery and mice were allowed to recover for an additional week prior to initiation of diet feedings.

### Diets

At 11 weeks of age, mice (Male, Female and OVX) were randomly assigned to either control purified AIN-76A low-fat diet (LFD) (3.77 kcal/g) or a purified high-fat diet (HFD) (40% of total kcals from fat; 4.57 kcal/g) designed to mimic the standard American diet (BioServ, Frenchtown, NJ, USA). We have used these diets in several of our previous studies [58–64]. Mice were initially provided the respective LFD or HFD for 20–21 weeks at which time baseline body composition (DEXA), microCT, and metabolic tests were performed. At the 20–21 week time point a group of mice (*n* = 8–9) were euthanized in order to collect blood and adipose tissue for baseline measurements of adipokines and adipose tissue gene expression. A separate group of mice (*n* = 15) received MC38 colon cancer cell injection (described below) at 20–21 weeks of diet treatment and continued their respective diets (LFD or HFD) for another 3 weeks. Thus, in total, these mice consumed the assigned diets for 23–24 weeks. This experiment was performed over 3 independent blocks, with *n* = 5 mice/group/block for a total of *n* = 15/group.

### Body weights, body composition and metabolism

Body weight was monitored on a weekly basis throughout the study. Body composition was assessed before cancer cell injection after 20–21 weeks of indicated diet feeding. For this procedure, mice were placed under brief anesthesia (isoflurane inhalation) and were assessed for lean mass, fat mass, body fat percentage and bone mineral density via dual-energy X-ray absorptiometry (DEXA) (Lunar PIXImus, Madison, WI). Metabolic parameters were assessed at 20–21 weeks of dietary treatment. After a five-hour fast, blood samples were collected from the tip of the tail. Blood glucose concentrations were determined in whole blood using a glucometer (Bayer Contour, Michawaka, IN). Collected blood was centrifuged at 4,000 rpm for 10 min at 4°C. Plasma was aliquoted and stored at –80°C until analysis. Plasma insulin (Mercodia Inc., Winston Salem, NC), high molecular weight (HMW) adiponectin (ALPCO), leptin (R&D systems), and insulin like growth factor-1 (R&D systems) concentrations were analyzed using commercial ELISA kit according to the manufacturer’s instructions. For glucose tolerance tests (GTTs) and insulin tolerance tests (ITTs), mice were fasted for 5 hours and glucose or insulin was administered (IP) at a dose of 2 g/kg or 0.75 U/kg of lean mass, respectively. Blood glucose concentrations (tail sampling) were measured intermittently over a two-hour period (0, 15, 30, 60, 90, and 120 minutes) for GTTs and intermittently over a one-hour period (0, 15, 30, 45, and 60 minutes) for ITTs using a glucometer (Bayer Contour, Michawaka, IN). For the ITT blood glucose levels are presented as % of baseline for each mouse. AUC was calculated using the trapezoidal rule.

### Micro computed tomography (CT) quantification of fat volume

Following 18 weeks of diet feeding, mice were placed under brief anesthesia (isoflurane inhalation) and positioned with both legs fully extended on the bed. The torso was scanned at an isotropic voxel size of 44 mm FOV (70 kVp, 114 uA) for 18 seconds using Quantum GX micro CT Imaging System (Perkin Elmer). Using, AccuCT micro CT analysis software, all scans were calibrated to Hounsfield Unites (HU) based on the standard densities of plastic (40) and air (-1000) plotted against the measured density at the time of scanning. All scans underwent low pass filter to remove background noise and cropped to a region of interest between the lumbar (L1 and L5) spine of each mouse. Threshold objects of bone (> 500 HU), soft tissue (75–500 HU) and adipose tissue (< 75 HU) were applied to all scans. Subcutaneous and visceral adipose tissue was differentiated using the abdominal wall as an anatomical landmark (adipose superficial to the soft tissue layer was marked subcutaneous and all adipose deep marked as visceral). Volumes of specified objects (bone, soft tissue, visceral fat and subcutaneous fat) were calculated using AccuCT micro CT analysis software. Volumes of adipose tissue were then normalized to the soft tissue volume calculated within each scan [65].

### Subcutaneous tumor model

MC38, murine colon adenocarcinoma cells (Kerafast; cat # ENH204-FP), were maintained in complete DMEM 4.5 g/L glucose with 10% heat inactivated fetal bovine serum (HI-FBS), 2 mM glutamine, and 1% penicillin/streptomycin. Cells were routinely passaged when 80% confluence was reached under sterile conditions and maintained in a 37°C, 5% CO2 incubator. Prior to injection, cells (passage < 20) were trypsinized, washed, counted with a hemocytometer and resuspended at 2 × 10^6^ cells/mL in phosphate buffered saline without Ca^++^ and Mg^++^. Mice were briefly anesthetized (isoflurane inhalation), and 100 μL of MC38 cell suspension (2 × 10^5^ cells/mouse) was injected subcutaneously using a 0.3 mL 29G syringe into the left dorsal lumbar region of each mouse. Viability of the cell suspension was confirmed > 95% using trypan blue staining before and after all injections were completed.

### Tissue collection

After 23–24 weeks of dietary treatment (3 weeks after tumor injection), mice were sacrificed via isoflurane inhalation for tissue collection. Given the association between CRC and leukocyte density, whole blood taken from the inferior vena cava was analyzed for hematology analysis using a VetScan Hm5 (Abaxis, Union City, CA, USA). The blood was spun at 4,000 RPM × 10 min and aliquoted plasma was stored at –80°C. Gonadal, mesentery, and peri-renal fat pads, as well as the spleen were removed, weighed, and immediately snap-frozen in liquid nitrogen and stored at −80°C until analysis. Subcutaneous tumors were removed, weighed and measured (length and width) using calipers. Half of the tumor was placed in complete DMEM to be used for cell sorting. The other half was snap frozen and stored at –80°C until analysis. For subcutaneous adipose tissue conditioned media collection, the dorsal lumbar portion of the inguinal fat pad was removed, briefly rinsed in PBS, cut into 3–5 mm pieces and placed into complete DMEM (4.5 g/L glucose with 10% HI-FBS, 2 mM glutamine, and 1% penicillin/streptomycin) at a concentration of 100 mg tissue/mL of media. Following 24 hr of rest/acclimation, the tissue was rinsed in PBS and placed in fresh complete DMEM with 2% HI-FBS. Conditioned media was collected after 24 hours, sterile filtered (0.22 uM syringe filter), and stored at –80°C until use.

### Flow cytometry and cell sorting

Tumors were mechanically disrupted in PBS with 5% HI-FBS using the gentle MACS dissociate and TAMs were isolated using FACS as described [66]. Briefly, tumor isolates underwent red blood cell (RBC) lysis and were filtered through a 100-μm then a 70-μm cell strainer prior to being stained with CD45-APC, F4/80-PE, and CD11b-FITC (Biolegend, San Diego, CA, USA) antibodies for 30 minutes. Following compensation using unstained and single stained controls, TAMs were quantified by gating for CD45+ cells followed by F480 and CD11b double positive gate. TAMs were sorted into a separate tube containing PBS with 5% HI-FBS and kept on ice until all samples were sorted. Data were acquired using a BD FACS Aria II cell sorter and analyzed using DIVA software. Freshly sorted TAMs were spun at 2000 RPM × 5 min, resuspended in Trizol reagent and stored at –20°C until RNA isolation.

### Quantitative real-time RT-PCR

RNA was isolated from FACS TAMs (~100,000/sample), and gonadal and subcutaneous adipose tissue depots using Trizol reagent. TaqMan reverse transcription reagents and gene expression assays (Applied Biosystems, Foster City, CA, USA) were used to reverse transcribe and to analyze the expression of the following genes: F4/80, CD11c, CD206, NOS, and ARG. Potential reference genes (18s, HMBS, TBP, B2M, H2AFV, and HPRT) were analyzed for stability using Qbase+ software (Biogazelle, Belgium) for each tissue analyzed. The optimal number of reference genes were determined by Qbase+ and the geometric mean of these genes was used as the normalization factor for each analysis: TAM (B2M and H2AFV), gonadal adipose tissue (H2AFV, 18S, HPRT, HMBS, TBP), and subcutaneous adipose tissue (B2M, TBP, HMBS) [64, 67]. Gene expression quantification was calculated using the ΔΔCT method and Qbase+ software. Values were normalized to the LFD female group.

### Statistical analysis

Data were analyzed using commercially available statistical software: Prism 7 (GraphPad Software, La Jolla, CA, USA). Outliers were identified from each data set using ROUT (Q = 1%) and removed prior to statistical analysis. A two-way ANOVA followed by a Tukey post-hoc analysis was used to determine differences between diet (HFD vs. LFD) and hormone status (female intact estrogen+/testosterone–; female OVX estrogen–/testosterone–; and male estrogen–/testosterone+). The relationship between tumor size and total fat was assessed using the Pearson correlation coefficient. Data are presented as the mean ± SEMs and the level of significance was set at *p* ≤ 0.05.

## Abbreviations

ARG: arginine
AUC: area under the curve
CRC: colorectal cancer
CT: computerized tomography
DEXA: dual-energy X-ray absorptiometry
FACS: fluorescence activated cell sorting
GTT: glucose tolerance test
HFD: high-fat diet
HMW: high molecular weight
IGF1: insulin-like growth factor-1
ITT: insulin tolerance test
IL-10: interleukin 10
LFD: low-fat diet
MCP1: monocyte chemoattractant protein 1
OVX: ovariectomized
RBC: red blood cell
TAM: tumor associated macrophage
TNF-α: tumor necrosis factor α
TGF-β: transforming growth factor β
WT: wildtype
